# Activation of Interleukin-32 Pro-Inflammatory Pathway in Response to Influenza A Virus Infection

**DOI:** 10.1371/journal.pone.0001985

**Published:** 2008-04-16

**Authors:** Wei Li, Yan Liu, Muhammad Mahmood Mukhtar, Rui Gong, Ying Pan, Sahibzada T. Rasool, Yecheng Gao, Lei Kang, Qian Hao, Guiqing Peng, Yanni Chen, Xin Chen, Jianguo Wu, Ying Zhu

**Affiliations:** State Key Laboratory of Virology, College of Life Sciences, Wuhan University, Wuhan, People's Republic of China; University of Birmingham, United Kingdom

## Abstract

**Background:**

Interleukin (IL)-32 is a recently described pro-inflammatory cytokine that has been reported to be induced by bacteria treatment in culture cells. Little is known about IL-32 production by exogenous pathogens infection in human individuals.

**Methods and Findings:**

In this study, we found that IL-32 level was increased by 58.2% in the serum samples from a cohort of 108 patients infected by influenza A virus comparing to that of 115 healthy individuals. Another pro-inflammatory factor cyclooxygenase (COX)-2-associated prostaglandin E2 was also upregulated by 2.7-fold. Expression of IL-32 in influenza A virus infected A549 human lung epithelial cells was blocked by either selective COX-2 inhibitor NS398 or Aspirin, a known anti-inflammatory drug, indicating IL-32 was induced through COX-2 in the inflammatory cascade. Interestingly, we found that COX-2-associate PGE_2_ production activated by influenza virus infection was significantly suppressed by over-expression of IL-32 but increased by IL-32-specific siRNA, suggesting there was a feedback mechanism between IL-32 and COX-2.

**Conclusions:**

IL-32 is induced by influenza A virus infection via COX-2 in the inflammatory cascade. Our results provide that IL-32 is a potential target for anti-inflammatory medicine screening.

## Introduction

Influenza A virus (IV) is a highly contagious single-stranded RNA virus that infects both the upper and lower respiratory tracts of humans. The host innate immune Toll-like receptor 3 (TLR3) was shown previously in cells of myeloid origin to recognize the viral replicative, intermediate double-stranded RNA (dsRNA). Thus, dsRNA is critical for the outcome of the infection and appears to be an active component of viral infection that stimulates antiviral activities. It accumulates during the replication of many viruses [1], [2], including influenza virus. Prominent sources of dsRNA include viral RNAs containing double-stranded secondary structure and dsRNA formed during viral replication [2]. Furthermore, TLR3 contributes directly to the immune response of respiratory epithelial cells to influenza A virus and dsRNA [3], [4]. Therefore dsRNA treatment was always used to mimic the viral infection in cell lines [5], [6], [7]. In macrophages, dsRNA and viral infection stimulate the expression of pro-inflammatory cytokines such as IL-1alpha, IL-1beta, tumor necrosis factor, and IL-6 [1], [8], [9], [10].

Cyclooxygenase (COX) is the rate-limiting enzyme in the biosynthetic pathway of prostaglandins and thromboxanes. Prostaglandins play an important role in many biological processes. Altered prostanoid production is associated with a variety of illnesses, including acute and chronic inflammation, cardiovascular disease and colon cancer [11]. Two isoforms of COX were described: COX-1 and COX-2. COX-1 is constitutively expressed in almost all tissues [12]; COX-2 is the inducible form of the enzyme, which is expressed in response to inflammatory stimuli and growth factors and is involved in mediating pain and inflammatory processes [13], [14]. In our previous studies, we identified two viral proteins, the nucleoprotein and spike protein of SARS-CoV, which were involved in up-regulating COX-2 [15], [16]. Prostaglandin E_2_ (PGE_2_) is synthesized from PGH_2_ in a variety of cells, which itself is synthesized from arachidonic acid by the enzyme prostaglandin synthetase COX-2. Its levels can be measured to access the COX-2 activity as described in reference [5], [6], [17], [18].

Interleukin-32 (IL-32), previously called natural killer cell transcript 4, has been recognized as a pro-inflammatory cytokine recently. It is mainly expressed in natural killer cells, T cells, epithelial cells and blood monocytes. IL-32 can induce the pro-inflammatory cytokines TNF-α and IL-1β in murine peritoneal macrophages as well as in phorbol ester-differentiated human THP-1 cells [19]. It plays an important role in innate and adaptive immune responses, synergism between IL-32 and other well-characterized players in innate immunity has recently been documented [20]. Furthermore, IL-32 contributes to the synovitis during rheumatoid arthritis [21]. It is stimulated by *Mycobacterium tuberculosis* [22] and it synergizes with NOD1 and NOD2 ligands to stimulate IL-1β and IL-6 release in a caspase-1-dependent manner [23]. Recently Proteinase 3 has been identified as a specific IL-32α binding protein which cleaves the cytokine to enhance its activity [24].

A number of investigations demonstrated that viral infections stimulate COX-2 expression, followed by PGE_2_ accumulation [6], [15], [16], [25], [26]. However, the role of influenza A virus infection in the regulation of the newly described pro-inflammatory factor IL-32 expression is still unclear. Furthermore, since COX-2 and IL-32 are obligatory mediators of inflammation, the question arises as to whether there is an interaction between the two proteins or they act as independent effectors of host inflammatory response to viral infection.

Because COX-2 and IL-32 gene expression are associated with inflammatory processes, the aim of this study is to investigate the role of influenza A virus infection in the regulation of IL-32 expression and to determine the molecular mechanisms responsible. Our results showed that influenza A virus infection or poly(IC) treatment activates COX-2 and IL-32 expression by a heretofore unrecognized mechanism, in which influenza A virus stimulates IL-32 expression through COX-2, and IL-32 feedback inhibits COX-2 expression.

## Methods

### Patients

The study examined 108 consecutive adults with influenza A virus infection (59 male, 49 female, aged 39.2±13.5 yr) seropositive for influenza A antigen and 115 healthy adults (62 male, 53 female, aged 37.6±11.3 yr) seronegative for influenza A antigen. All adults were seronegative for markers of hepatitis B virus (HBV), hepatitis C virus (HCV), hepatitis Delta virus, and HIV. All of the investigated serum samples were obtained with the help from Hubei provincial Center for Disease Control and Prevention (Hubei CDC). Informed consent was obtained from each of the patients. The collection of blood samples for research was approved by the Institutional Review Board of the College of Life Sciences, Wuhan University in accordance with guidelines for the protection of human subjects.

### Virus

The influenza virus strain A/chicken/Hubei/327/2004 (H5N1) used in this study was provided by China Center for Type Culture Collection (CCTCC). Stock virus was propagated in 10-day-old embryonated chicken eggs for 36 to 48 h at 37°C. The allantoic fluid was then harvested, and aliquots were stored at –80°C before being used. The final concentration of H5N1 virus infection used in this study was 1 multiplicity of infection (MOI).

### Plasmids, antibodies, and inhibitors

pcDNA3.1-COX-2 was a gift from Dr. Kenneth K. Wu (University of Texas-Houston Medical School, Houston, Texas, USA). Luciferase reporter vector (pGL3) containing a COX-2 promoter region (−891/+9) was constructed previously [27]. IL-32 promoter (−746/+25) was amplified from human genomic DNA by PCR, using the following primers: 5′-GTTACGCGTCCTATTTCAATATGACTGGT-3′ (sense), 5′- GTTAAGCTTAGGGAAAGTCCAGACTCGGG -3′ (antisense); and then inserted into pGL3-Basic vector to generate IL-32 promoter and Luciferase gene fusion plasmid (pIL-32-Luc). An IL-32 construct was created by RT-PCR amplification of the open reading frame from A549 human lung epithelial cells. To create IL-32-encoding vector, the IL-32 beta gene was amplified using the following primers: 5′- CATGAATTCCATGTGCTTCCCGAAGG -3′ (sense), 5′- CTACTCGAGGTATCTTCATTTTGAGGATTG -3′ (antisense); in which *Eco*RI and *Xho*I sites were introduced, respectively. The PCR product was cloned into *Eco*RI and *Xho*I sites of pCMV-tag2A (Stratagene) to generate plasmid Flag2A-IL-32, in which IL-32 beta protein was tagged by FLAG.

All of the ten genes of influenza A virus H5N1 were obtained by RT-PCR from H5N1 infected A549 cells and constructed into pCMV-Flag2A vector to generate IV-gene expressing plasmids (Flag2A-HA, Flag2A-NA, Flag2A-NP, Flag2A-NS1 Flag2A-NS2, Flag2A-M1, Flag2A-M2, Flag2A-PA, Flag2A-PB1, Flag2A-PB2) using specific pairs of primers for each influenza A virus gene as follows: HA: 5′- ACGGGATCCGATGGAGAAAATAGTGCTT -3′ (sense), 5′- ACGCTCGAGTGAACTCACAAATTTA -3′ (antisense); NA: 5′-GCGGAATTCTATGAATCCAAATCAGAAGATAATAAC-3′ (sense), 5′- AAAGGGCCCCTACTTGTCAATGGTGAATGG -3′ (antisense); NP: 5′- AAAGGATCCCATGGCGTCTCAAGGCACC -3′ (sense), 5′- ACCGAATTCTTAATTGTCATACTCC -3′ (antisense); NS1: 5′- ACAGGATCCGATGGATTCCAACACTGTGTC -3′ (sense), 5′- CTTGAATTCTCAGCCACCTTATTTC -3′ (antisense); NS2: 5′- ACAGGATCCGATGGATTCCAACACTGTGTC -3′ (sense), 5′- CTAGGGCCCATCATTAAATAAGCTGAAACGA -3′ (antisense); M1: 5′- TCTGAATTCGATGAGTCTTCTAAC -3′ (sense), 5′- TTAGGGCCCGCAACAACAAGAGGATCACT -3′ (antisense); M2: 5′- TCTGAATTCGATGAGTCTTCTAAC -3′ (sense), 5′- TTTCTCGAGGGTAGTTTTTTACTCCAAT -3′ (antisense); PA: 5′- AATGGATCCTATGGAAGACTTTGTGCGAC -3′ (sense), 5′- CAGGGGCCCTACTTCAGTGCATGTGCGAG -3′ (antisense); PB1: 5′- CAAACGGATCCCATGGATGTCAATCCGAC -3′ (sense), 5′- CAAGAAGCTTTCACTATTTCTGCCGTCTG -3′ (antisense); PB2; 5′- GCAAAAGCAGGTCAAATATA -3′ (sense), 5′- GTTTTTAAACAATTCGACACTA -3′ (antisense); IL-32 siRNA plasmid was constructed by ligating the corresponding pairs of oligonucleotide to p*Silencer™* 2.1-U6 neo (Ambion, Inc., Augstin, TX, USA) based on the sequences described previously [28]. All constructs was confirmed by DNA sequencing.

Monoclonal mouse antibody against human COX-2 was purchased from Cayman Chemical Company (Ann Arbor, MI, USA). Polyclonal goat antibody against human IL-32 was purchased from R&D Systems, Inc. USA. Polyclonal goat antibody specific for human β-actin (SC-1616) were purchased from Santa Cruz Biotechnology, Inc (Santa Cruz, CA, USA).

N-(2-cyclohexylosy-4-nitrophenyl)-methanesulphonamide (NS398) (Promega, Madison, WI) was dissolved in DMSO and used as indicated concentrations according to reference [29]. Aspirin (Sigma, St. Louis, MO, USA), Synthetic polyinosinic-polycytidylic acid poly(IC) (Sigma, St. Louis, MO, USA), and recombinant human interferon-γ (IFN-γ) (Peprotech, London, UK) were dissolved in PBS and used at a final concentration of 5 mM, 50 µg/ml, 150 U/ml, respectively.

### Cell culture

HEK 293T cells were cultured in DMEM (GibcoBRL, USA), human lung epithelial cells A549 were cultured in F12K media (GibcoBRL, USA), respectively. All media were supplemented with 10% fetal calf serum, 100 U/ml penicillin and 100 µg/ml streptomycin sulfate and all cell cultures were maintained at 37°C in a 5% CO_2_ incubator.

### Transient transfection and Luciferase reporter gene assays

Cells were plated at density of 4.0×10^5^ cells per 24-well plate or 6-well plate and grown to confluence reaching about 80% at the time of transfection. The plasmids which express COX-2, IL-32, the pGL3-promoter plasmids, pRL-TK (Promega) were co-transfected into the cells by using Lipofectamine 2000 reagent (Invitrogen). If necessary, poly(IC), IFN-γ, NS398, and Aspirin were added into the culture media after transfection. 24 h post-transfection, cells were serum-starved for another 24 h before being harvested. Luciferase activities were measured 48 h after transfection according to the manufacturer's instructions (Promega) and Renilla Luciferase activities were determined as internal control for transfection efficiency as previously described [15]. Assays were performed in triplicate and expressed as means±s.d. relative to vector or mock control as 100 (RLU) or luciferase activity (LUC).

### Semiquantitative RT-PCR analysis

Total RNA, isolated from A549 cells using Trizol reagent (Invitrogen, Carlsbad, CA, USA), was treated with DNase I and reverse-transcribed with MLV reverse transcriptase (Promega) and random primers (Takara). PCR was performed in 25 µl reactions with the detection primer pairs described as follows: IL-32 sense: 5′- CATGAATTCCATGTGCTTCCCGAAGG-3′; IL-32 antisense: 5′- CTACTCGAGGTATCTTCATTTTGAGGATTG -3′. Detection primers for COX-2, and β-actin were described previously [15]. β-actin was amplified by PCR for normalization in all reactions. The primers for IL-32 were designed for detecting all known isoforms of human IL-32. PCR products were analyzed by electrophoresis on 1% agarose gel containing ethidium bromide.

### Western blot analysis

Protein extracts of cultured cells were prepared by suspending cells in lysis buffer (0.01% EDTA, 0.1% Triton X-100, 10% proteinase inhibitors cocktail), sonication, and centrifugation. Concentrations of proteins in supernatant were quantified using protein assay kit (Bio-Rad). Western blot analysis was performed using COX-2 and IL-32 antibodies and sample loading was normalized by using β-actin antibody. Immunoblots were visualized with the ECL detection system (Pierce, Rockford, IL, USA).

### COX-2 activity

Because increased PGE_2_ is the metabolite of COX-2 enzyme catalysis in epithelial cells, COX-2-derived PGE_2_ levels in the culture medium was assayed by the Biotrak Prostaglandin E2 Enzyme Immunoassay system (R & D Systems) according to the manufacturer's protocol.

### ELISA for IL-32 measurement

ELISA assays were developed using IL-32 antibody and a polypeptide, H-KEELTPQKCSEPQSSK-OH, (GL Biochem Ltd. Shanghai China) was synthesized as an antigen for making an IL-32 protein standard curve. ELISAs were prepared by coating the bottom of a 96-well plate with diluted serum samples or culture media. 50 µl of 2 µg/ml capture antibody was incubated for one hour then washed three times with TBST, followed by HRP-labeled secondary antibody incubation for 15 minutes, and finally washed six times with TBST. ELISAs were developed with TMB (Sigma) substrate and the absorbance at double-wavelength 450 nm/630 nm was measured. IL-32 concentrations were calculated from the standard curve.

### Statistical analysis

All experiments were reproducible and were carried out in triplicate or quadruplicate. Each set of experiments was repeated at least three times with similar results, and a representative one is shown. The results are presented at the means±s.d. Student's *t* test for paired samples was used to determine statistical significance. Differences were considered statistically significant at a value of *P*≤0.05.

## Results

### Serum levels of PGE_2_ and IL-32

The serum levels of PGE_2_ and IL-32 were significantly higher in the cohort of patients with influenza A virus infection investigated in comparison with healthy control individuals (mean±SEM for PGE_2_, 808.7±52.9 vs. 299.5±45.4 pg/ml; for IL-32, 183.8±43.6 vs. 116.2±29.3 pg/ml, respectively, *P*<0.01 Mann Whitney-U) as described in Table 1.

**Table 1:** Demographic and baseline characteristics, serum levels of PGE_2_ and IL-32 from influenza A positive patients and healthy individuals.

Characteristic	Healthy Individuals (N = 115)	Patients (N = 108)	P Value
Age-yr	37.6±11.3	39.2±13.5	0.85
Male sex-no.(%)	62 (54)	59 (55)	0.91
Race or ethnic group-no.(%) Asian	115 (100)	108 (100)	1.00
Time-no.(%)	115 (100)	108 (100)	1.00
Oct.8th/2007-Oct.19th/2007			
Region-no.(%)	115 (100)	108 (100)	1.00
Hubei province, China			
HA-Antigen-positive-no.(%)	0 (0)	108 (100)	<0.001
Anti-HA-positive-no.(%)	0 (0)	108 (100)	<0.001
Viral genotype A (H3/H1)-no.	0	108 (85/23)	
PGE2-pg/ml	299.5±45.4	808.7±52.9	<0.001
IL-32-pg/ml	116.2±29.3	183.8±43.6	<0.001

### COX-2 and IL-32 activation in response to influenza A virus infection and dsRNA treatment in a time-dependent manner

**Figure 1:**
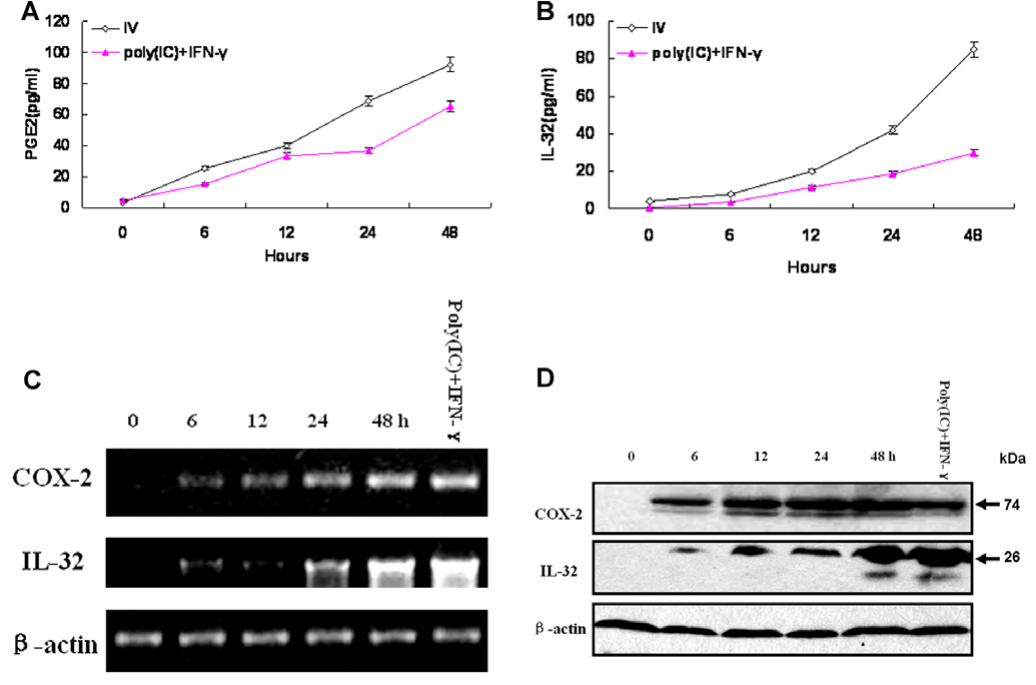
COX-2 and IL-32 were induced in A549 human lung epithelial cells in response to influenza A virus infection or dsRNA treatment in a time-dependent manner. Time-dependent of PGE_2_ (A) and IL-32 (B) accumulations in A549 culture supernatants are shown in response to influenza A virus infection (1 MOI) or poly(IC) (50 µg/ml)+IFN-γ (150 U/ml) treatment. Data are expressed as mean±s.d. of three independent experiments. Time-dependent of COX-2 and IL-32 mRNA accumulations (C) in cell lysates are shown by Semiquantitative RT-PCR analysis and protein productions (D) shown by western blot analysis, in which A549 cells were harvested at indicated time points after influenza A virus infection (1 MOI) or 48 h treatment with poly(IC) (50 µg/ml)+IFN-γ (150 U/ml). The gel or blot is a representative of three experiments with similar results.

It has been reported that influenza virus can activate the expression of COX-2 in cell culture systems [26] and that dsRNA can induce the production of COX-2, followed by PGE_2_ release [5]. Initially, we investigated whether influenza A virus infection and dsRNA plays a role, if any, in the regulation of IL-32 expression. A549 cells were infected with influenza A virus or treated with poly(IC)+IFN-γ, results showed that A549 cells were susceptible to influenza virus infection and apparent cytopathic effect was observed after viral infection. The culture supernatants and cell lysates were harvested at different time points after infection or induction as follows: 0 h, 6 h, 12 h, 24 h, 48 h. PGE_2_ accumulation (Fig. 1A) and IL-32 production (Fig. 1B) in culture supernatants were measured as described in materials and methods section. COX-2, IL-32 mRNA (Fig. 1C) and protein (Fig. 1D) expression levels in lysated cells were examined by semiquantitative RT-PCR and western blot analyses, respectively. These results suggested that both influenza A virus infection and dsRNA treatment could activate the expression of COX-2 and IL-32 in A549 cells in a time-dependent manner.

### Identification of the viral components involved in influenza A virus-stimulated COX-2 and IL-32 expression

**Figure 2:**
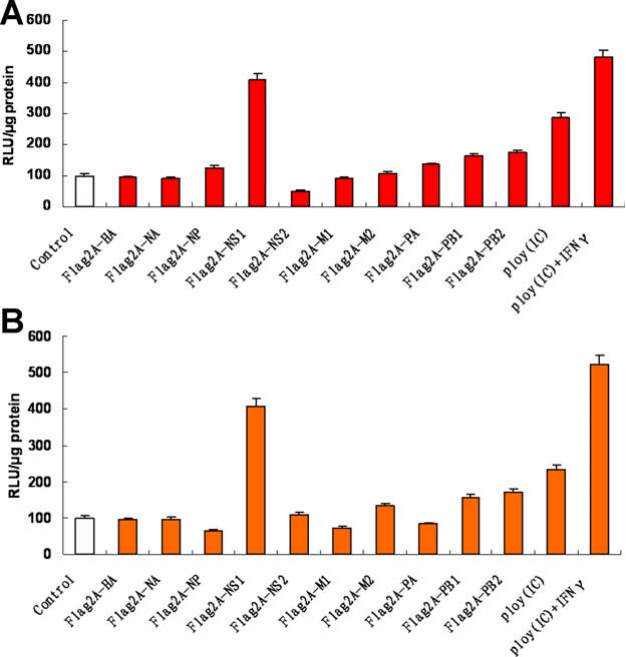
Screening of viral proteins or viral replicative intermediate dsRNA involved in induction of COX-2/ IL-32. Reporter plasmids pCOX-2-Luc (A), pIL-32-Luc (B), and pRL-TK were cotransfected along with all 10 viral gene constructs (Flag2A-HA, Flag2A-NA, Flag2A-NP, Flag2A-NS1, Flag2A-NS2, Flag2A-M1, Flag2A-M2, Flag2A-PA, Flag2A-PB1, Flag2A-PB2) and control vectors (Flag2A) or treated with or without poly(IC) (50 µg/ml), poly(IC) (50 µg/ml)+IFN-γ (150 U/ml) into A549 cells which are indicated on the horizontal axis respectively. Luciferase activity was measured as described in methods section. Results are expressed as the mean±s.d. of three independent experiments performed in triplicate and normalized by Renilla activities.

To identify the viral components which play important roles in IV-stimulated pro-inflammatory factors COX-2 and IL-32 expression, we screened all ten proteins of influenza virus: HA, NA, NP, NS1, NS2, M1, M2, PA, PB1, PB2 and poly(IC) (to mimic viral replicative intermediate dsRNA) by luciferase assays. Results showed that poly(IC), poly(IC)+IFN-γ, and NS1 are the most important factors in the induction of either COX-2 (Fig. 2A) or IL-32 (Fig. 2B) promoter activities, which means dsRNA and NS1 are the key viral components involved in IV-triggered COX-2 and IL-32 expression during viral infection. In this study, we focused on the function of dsRNA in inflammatory response to IV infection.

### DsRNA stimulates IL-32 through COX-2 pathway during inflammatory response

To define the role of COX-2 in the regulation of influenza A virus induced pro-inflammatory factor IL-32, poly(IC)+IFN-γ treatment was used to mimic the influenza A virus infection in following experiments as reported previously[30].

**Figure 3:**
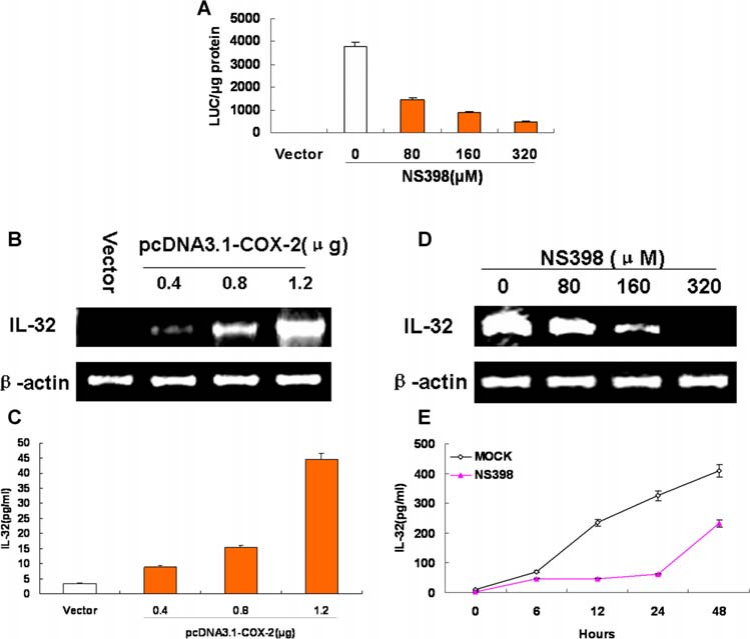
DsRNA induces IL-32 is in COX-2-dependent manner. Reporter plasmid pIL-32-Luc (A), and pRL-TK were cotransfected along with pcDNA3.1-COX-2 or pcDNA3.1 into A549 cells. Transfected cells by COX-2 plasmids were incubated for 12 h and then maintained for 36 h with different final concentrations of NS398 as indicated, respectively. Luciferase activity was measured. Results are expressed as the mean±s.d. of three independent experiments performed in triplicate and normalized by Renilla activities. A549 cells were transfected with different indicated amounts of pcDNA3.1-COX-2 or pcDNA3.1. RT-PCR for IL-32 and β-actin (internal control) (B) in cell lysates, Elisa for IL-32 (C) in culture supernatants were performed. (D) A549 cells were treated with poly(IC) (50 µg/ml)+IFN-γ (150 U/ml) for 12 h and then different concentration of NS398 was added as indicated for another 36 h. IL-32 and β-actin mRNA level were examined by RT-PCR. (E) A549 cells were stimulated by poly(IC) (50 µg/ml)+IFN-γ (150 U/ml) with or without 80 µM NS398 for 48 h. Time-dependent of IL-32 expression in culture supernatants were measured. The data represent mean±s.d. of three separate experiments.

First, the effects of COX-2 on the activation of IL-32 promoter were determined. A549 cells were cotransfected with the reporter plasmid pIL-32-Luc and pcDNA3.1, pcDNA3.1-COX-2 plus different concentration of NS398 as mentioned in Fig. 3A, pcDNA3.1 was used as empty vector control. Results from Luciferase activity assay showed that the level of IL-32 promoter activity was increased by COX-2 over-expression and it was blocked by COX-2 inhibitor NS398 in a dose-dependent manner.

Secondly, to determine the effects of COX-2 on the activation of IL-32 mRNA level and protein expression, A549 cells were transfected with different amounts of pcDNA3.1-COX-2. Results from RT-PCR using IL-32-specific, or β-actin-specific primers showed that the levels of IL-32 mRNA were increased as the amount of pcDNA3.1-COX-2 increased, but the levels of β-actin mRNA remained relatively constant (Fig. 3B). Furthermore, IL-32 production in culture supernatants was stimulated by COX-2 over-expression in a dose-dependent manner (Fig. 3C).

Thirdly, results from RT-PCR analyses showed that the level of IL-32 mRNA activated by poly(IC)+IFN-γ was suppressed by COX-2-specific inhibitor NS398 in a dose dependent manner (Fig. 3D). Furthermore, IL-32 accumulation in culture supernatants stimulated by poly(IC)+IFN-γ were suppressed by 80 µM NS398 (Fig. 3E).

Taken together, these data suggest that COX-2 is an upstream regulatory factor of dsRNA-triggered IL-32 production.

### IL-32 feedback inhibits dsRNA-induced COX-2 expression

**Figure 4:**
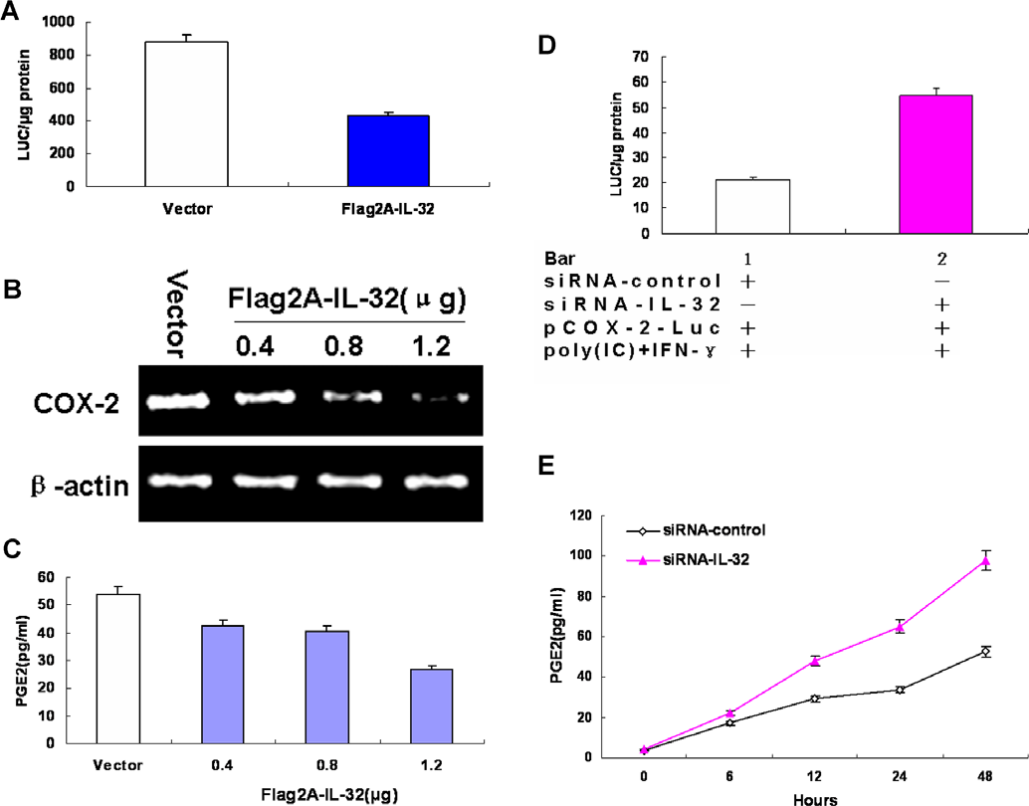
The feedback inhibition of IL-32 to dsRNA-induced COX-2 expression. Reporter plasmid pCOX-2-Luc (A) and pRL-TK were cotransfected along with Flag2A-IL-32 or control vectors (Flag2A) into A549 cells. Luciferase activity was then measured 48 h post transfection. A549 cells were transfected with different amounts of Flag2A-IL-32 as indicated or Flag2A and stimulated by poly(IC) (50 µg/ml)+IFN-γ (150 U/ml) for 48 h. RT-PCR for COX-2 and β-actin (B) in lysated cells, measurement of PGE_2_ release (C) in culture supernatants were taken. Reporter plasmid pCOX-2-Luc (D) and pRL-TK were cotransfected along with siRNA-IL-32 or siRNA-control into A549 cells and treated with poly(IC) (50 µg/ml)+IFN-γ (150 U/ml) for 48 h. Luciferase activity was then measured. (E) A549 cells were transfected with siRNA-IL-32 or siRNA-control and stimulated by poly(IC) (50 µg/ml)+IFN-γ (150 U/ml) for 48 h. Time-dependent PGE_2_ release in culture supernatants was measured. The data represent mean±s.d. of three separate experiments.

The effect of IL-32 on the regulation of COX-2 promoter was determined. A549 cells were cotransfected with the reporter plasmid pCOX-2-Luc and pCMV-Flag2A, Flag2A-IL-32. Results from Luciferase activity assay showed that the level of COX-2 promoter activity was decreased by IL-32 over-expression (Fig. 4A).

To determine the effects of IL-32 on the regulation of COX-2 mRNA expression, PGE_2_ production, A549 cells were transfected with different amounts of Flag2A-IL-32 and treated with poly(IC)+IFN-γ as the inducer. Results from RT-PCR using COX-2-specific, or β-actin-specific primers showed that the levels of COX-2 mRNA was decreased as the amount of Flag2A-IL-32 increased, but the levels of β-actin mRNA remained relatively constant (Fig. 4B). Furthermore, PGE_2_ production in culture supernatants was suppressed by IL-32 over-expression in a dose-dependent manner (Fig. 4C).

To confirm the above results, IL-32-specific siRNA was cotransfected along with reporter plasmid pCOX-2-Luc into A549 cells and treated with poly(IC)+IFN-γ. Results from Luciferase activity assay showed that the level of COX-2 promoter activity was increased by knocking down IL-32 (Fig. 4D). Furthermore, PGE_2_ production in culture supernatants stimulated with poly(IC)+IFN-γ was enhanced by knocking down IL-32 (Fig. 4E).

Taken together, these data suggest that IL-32 plays a very important role in the inflammatory response following an influenza A/COX-2/IL-32 dependent positive regulatory order, while a negative feedback to COX-2 biosynthesis was also first observed.

### Influenza A-induced PGE_2_ production and IL-32 expression were inhibited by NS398 and Aspirin

**Figure 5:**
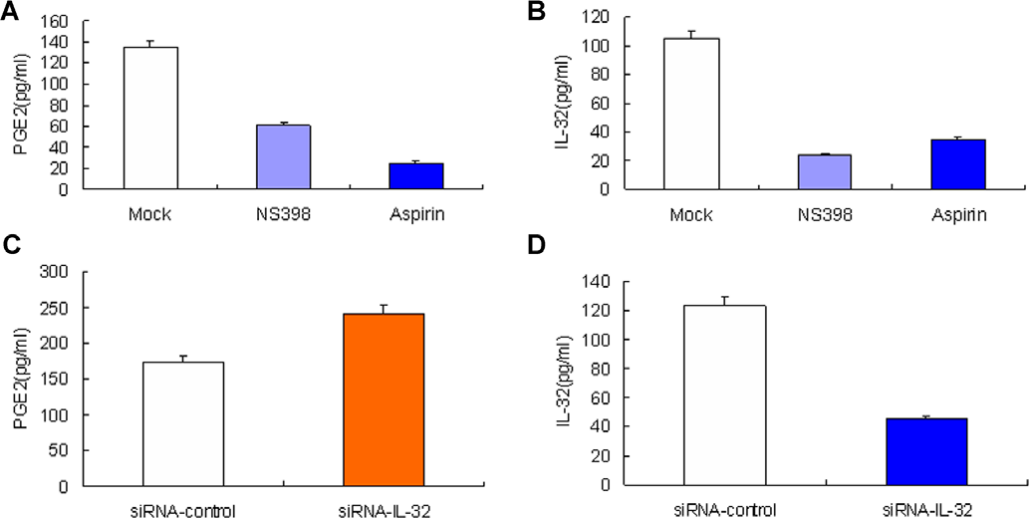
Selective COX-2 inhibitor NS398 suppresses IL-32 expression and IL-32-specific siRNA upregulates COX-2 in influenza A virus infected A549 cell. A549 cells were infected by influenza A virus (1 MOI) and treated with or without 80 µM NS398, 5 mM Aspirin for 48 h as indicated on the horizontal axis. PGE_2_ release (A) and IL-32 production (B) in culture supernatants were measured. A549 cells were transfected with siRNA-IL-32 or siRNA-control and infected by influenza A virus (1 MOI) for 48 h. PGE_2_ release (C) and IL-32 production (D) in culture supernatants were measured. The data represent mean±s.d. of three separate experiments.

To confirm the COX-2 and IL-32 regulatory loop, A549 cells were infected by influenza A virus and treated with or without selective COX-2 inhibitor NS398, or non-selective COX inhibitor Aspirin, a widely used anti-inflammatory drug. Results showed that IV-triggered PGE_2_ release (Fig. 5A) and IL-32 (Fig. 5B) production in the culture supernatants were inhibited by either NS398 or Aspirin, which indicated that IL-32 is an important pro-inflammatory factor downstream of COX-2 during influenza A virus infection.

### Influenza A-induced PGE_2_ production was increased by knocking down IL-32

The effect of IL-32 on the regulation of IV infection induced PGE_2_ production was also determined. We found that PGE_2_ release in A549 cell culture supernatants stimulated by IV infection was increased (Fig. 5C) and IL-32 production decreased (Fig. 5D) by knocking down IL-32.

**Figure 6:**
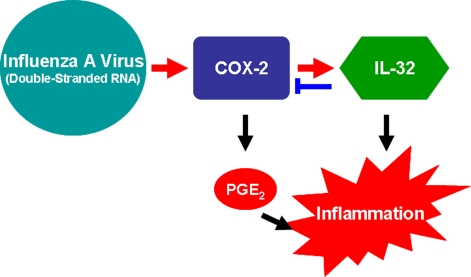
A model for regulation of pro-inflammatory factors COX-2 and IL-32 expression in response to influenza A virus infection and double-stranded RNA

Taken together, these data demonstrate that a novel inflammatory pathway in response to influenza A virus infection and dsRNA treatment was identified as described in Fig. 6.

## Discussion

Our results demonstrate that significant increase of serum COX-2-derived PGE_2_ and IL-32 levels were observed in influenza A infected patients compared with healthy individuals. Viral infection resulted in 2.7-fold increase in PGE_2_ synthesis and 58.2% increase in IL-32 production. Both influenza A virus infection and poly(IC)+IFN-γ treatment in A549 human lung epithelial cells were able to induce COX-2/ IL-32 mRNA and protein expression as well as PGE_2_ and IL-32 accumulation in the cell culture supernatants. IL-32 was induced by influenza virus infection through COX-2-dependent mechanism.

Although pro-inflammatory factor COX-2 has been identified as an obligatory mediator in the airway inflammation during influenza virus infection [26], virtually little is currently known regarding the regulation of a newly identified proinfammatory factor IL-32 in influenza A virus infection or the mechanism whereby influenza A virus upregulates IL-32 expression. The present study provides considerable new information relevant to these issues. We identified first a COX-2/IL-32 regulatory loop in which COX-2 upregulated IL-32 expression and IL-32 feedback inhibited COX-2 expression in influenza A virus infected A549 lung epithelial cells. At present, a possible “cross-talk” between two important pro-inflammatory factors nitric oxide synthase (NOS) and COX has been extensively examined in different cell lines, tissues and under different pathophysiological conditions with conflicting results [31]. But the relationship between COX-2 and IL-32 has not been evaluated.

Comparing the IV infection induced IL-32 level in cell culture with that in human individuals, a significant difference between them was observed, over 10-fold increase in cell culture versus 58.2% in human individuals. It is likely the pro-inflammatory factor is not able to be increased sharply under physiological conditions due to complex inflammation network in which pro-inflammatory factors regulate each other.

Two viral components, the NS1 and viral replicative intermediate dsRNA were identified to be involved in IV infection triggered COX-2 and IL-32 expression in A549 cells. Because TLR3 contributes directly to the inflammatory response of respiratory epithelial cells to both influenza A virus and dsRNA, we focused on the function of dsRNA in the regulation of COX-2 and IL-32 expression in this study. Role of viral NS1 protein in the pro-inflammatory process need further investigation.

Cyclooxygenase metabolizes arachidonic acid to prostaglandins (PGs) and thromboxane [32]. COX-2 has been elucidated to be crucial to the inflammatory response [33] and tumorigenesis [34], [35], so nonsteroidal anti-inflammatory drugs (NSAIDs) selective for its inhibition are used clinically to treat inflammatory arthropathies but always cause many side effects such as gastrointestinal (GI) mucosa damage and cardiovascular adverse events [36], [37], [38]. Aspirin is one of the classic NSAIDs, and its anti-inflammatory activity is associated with cyclooxygenase inhibition directly or indirectly [39]. NSAID-associated GI mucosal injury is an important clinical problem and the increased knowledge of physiological roles of COX-2 enzyme in a variety of tissues, including stomach and kidney, together with the withdrawal from the clinic trials of rofecoxib and valdecoxib because of cardiovascular toxicity, have challenged the benefits of selective COX-2 inhibition [38]. Our study identified a novel COX-2 downstream pro-inflammatory factor IL-32 which could be a potential target for screening anti-inflammatory drugs with less adverse effects.

In summary, these studies provide new insights into a novel model as described in Fig. 6 in which influenza A virus or poly(IC)+IFN-γ triggers pro-inflammatory factors COX-2 and IL-32 expression, COX-2-derived PGE_2_ production, and subsequent host inflammatory responses. In this model, we further demonstrate that COX-2 is located up-stream of IL-32 in the positive regulatory pathway. The cross-talk between the two genes is described as follows: COX-2 upregulates IL-32 production, and conversely, IL-32 attenuates COX-2 activity.
